# Local Induction of Immunosuppressive CD8^+^ T Cells in the Gut-Associated Lymphoid Tissues

**DOI:** 10.1371/journal.pone.0015373

**Published:** 2010-10-20

**Authors:** Diana Fleissner, Wiebke Hansen, Robert Geffers, Jan Buer, Astrid M. Westendorf

**Affiliations:** 1 Institute of Medical Microbiology, University Hospital Essen, University Duisburg-Essen, Essen, Germany; 2 Department of Cell Biology, Helmholtz Centre for Infection Research, Braunschweig, Germany; New York University, United States

## Abstract

**Background:**

In contrast to intestinal CD4^+^ regulatory T cells (T_regs_), the generation and function of immunomodulatory intestinal CD8^+^ T cells is less well defined. To dissect the immunologic mechanisms of CD8^+^ T cell function in the mucosa, reactivity against hemagglutinin (HA) expressed in intestinal epithelial cells of mice bearing a MHC class-I-restricted T-cell-receptor specific for HA was studied.

**Methodology and Principal Findings:**

HA-specific CD8^+^ T cells were isolated from gut-associated tissues and phenotypically and functionally characterized for the expression of Foxp3^+^ and their suppressive capacity. We demonstrate that intestinal HA expression led to peripheral induction of HA-specific CD8^+^Foxp3^+^ T cells. Antigen-experienced CD8^+^ T cells in this transgenic mouse model suppressed the proliferation of CD8^+^ and CD4^+^ T cells *in vitro*. Gene expression analysis of suppressive HA-specific CD8^+^ T cells revealed a specific up-regulation of CD103, Nrp1, Tnfrsf9 and Pdcd1, molecules also expressed on CD4^+^ T_reg_ subsets. Finally, gut-associated dendritic cells were able to induce HA-specific CD8^+^Foxp3^+^ T cells.

**Conclusion and Significance:**

We demonstrate that gut specific antigen presentation is sufficient to induce CD8^+^ T_regs_ *in vivo* which may maintain intestinal homeostasis by down-modulating effector functions of T cells.

## Introduction

Tolerance to self antigens is crucial to maintain intestinal homeostasis and to avoid autoimmunity in the gut. Several mechanisms contribute to peripheral tolerance, including clonal deletion, clonal anergy and immune suppression by T_regs_. The most prominent population of T_regs_ belongs to the CD4^+^ T cell subset. Much less attention has been given to the inhibitory capacity of CD8^+^ T cells. Nevertheless, a role for CD8^+^ T cells in the *in vivo* suppression of self-reactive T cells has also been described [1]. Although a number of CD8^+^ T cell clones with inhibitory activity have been reported [2]–[5], the nature of primary CD8^+^ T_regs_ and the mechanisms underlying their generation remain elusive.

Different CD8^+^ T_reg_ populations are thought to be involved in control of mucosal immune responses. Mayer and coworkers suggested that the deficiency of CD8^+^ T_regs_ in the lamina propria (LP) may lead to the development of inflammatory bowel disease (IBD) [6], [7]. CD8^+^ T cells isolated from noninflamed mucosa displayed suppressive capabilities; in contrast, LP CD8^+^ T cells derived from patients suffering from IBD were not able to suppress immune responses. In addition, Ménager-Marcq et al. have demonstrated that CD8^+^CD28^−^ but not CD8^+^CD28^+^ T cells freshly isolated from the spleen or the gut efficiently prevented the development of colitis in an adoptive transfer model where the injection of CD45RB^high^ into RAG2-deficient mice led to intestinal inflammation [8]. Very recently, a population of CD8^+^CD25^+^Foxp3^+^ T_regs_ was detected in the blood and even more prominently augmented in colorectal tumor tissues of patients suggesting that these cells may contribute to tumor-driven immune escape in the intestinal mucosa [9]. In a previous study we could demonstrate that the intestinal expression of a self antigen led to the peripheral induction of antigen-specific CD8^+^Foxp3^+^ T cells *in vivo* [10].

For CD4^+^ T_regs_ it has been suggested that dendritic cells (DCs) located in the intestine are able to induce T_regs_ [11]. New studies support this idea by showing that the catalysis of vitamin A into retinoic acid (RA) in gut-associated DCs enhances the TGF-β-dependent conversion of naïve T cells into T_regs_ and also directs homing to the gut [12], [13]. In the present study we dissected the phenotype and function of induced CD8^+^ T_regs_ and studied the induction of CD8^+^ T_regs_ in the intestinal mucosa.

## Materials and Methods

### Mice

VILLIN-HA mice express the hemagglutinin (HA) from influenza virus A/PR8/34 under control of the enterocyte-specific villin promoter [14], [15]. CL4-TCR transgenic mice express an α/β-TCR which recognizes an epitope of the HA protein presented by MHC class I (the H-2K^d^:HA512-520 complex) [16]. For initial experiments VILLIN-HA transgenic mice were crossed with CL4-TCR transgenic animals. Foxp3/GFP mice express both forkhead box P3 (Foxp3) and green fluorescent protein (GFP) under the endogenous regulatory sequence of the Foxp3 locus and were obtained from Charles River Laboratories. For the isolation of HA-specific CD8^+^Foxp3^+^ T cells CL4-TCR transgenic mice were crossed to Foxp3/GFP reporter mice. TCR-HA transgenic mice expressing an α/β-TCR recognizing the MHC class II (H-2E^d^:HA_110–120_)-restricted epitope of the HA protein have been described previously [17]. BALB/c mice were obtained from Harlan. All animal experiments were performed in accordance with institutional, state and federal guidelines (approved by the Landesamt für Natur, Umwelt und Verbraucherschutz Nordrhein-Westfalen, Az. 8.87–50.10.34.08.092).

### Antibodies and flow cytometry

All antibodies were obtained from BD Biosciences. α-Foxp3 staining (eBioscience) was performed according to the manufacturer's recommendations. For identification of HA-specific CD8^+^ T cells, cells were stained with APC-conjugated recombinant MHC class I pentamers H-2K^d^IYSTVASSL (Proimmune). The monoclonal antibody 6.5 (α-TCR-HA) was purified from hybridoma supernatant. Flow cytometric analyzes were done on a FACSCalibur with the CellQuest software (BD Biosciences).

### Cytometric bead array

Quantification of cytokines in culture supernatants was performed using the cytometric bead array kit according to the manufacturer's instructions (BD Bioscience). Data acquisition was performed by flow cytometry using a FACSCalibur. Acquired data were analysed using BD Bioscience Cytometric Bead Array software.

### DNA micorarrays

Total RNA from 10^6^ sorted CD8^+^ T cells was isolated using the RNAeasy kit (Qiagen). Quality and integrity of total RNA was assessed by running all samples on an Agilent Technologies 2100 Bioanalyser (Agilent Technologies). 12.5 µg of each biotinylated cRNA preparation was fragmented and placed in a hybridization cocktail containing four biotinylated hybridization controls (BioB, BioC, BioD, and Cre) as recommended by the manufacturer. Samples were hybridized to an identical lot of Affymetrix MOE430 2.0 for 16 hours. After hybridization the GeneChips were washed, stained with SA-PE and read using an Affymetrix GeneChip fluidic station and scanner. Analysis was done with gene expression software GCOS 1.2 (Affymetrix) and Genesis 1.6.

### Real-time RT PCR

Total RNA was prepared from isolated CD8^+^ T cells using the RNeasy kit (Qiagen) following cDNA synthesis by Superscript II Reverse Transcriptase (Invitrogen) and OligodT mixed with Random Hexamer primers according to the manufacturer's recommendations. Real-time RT PCR was done in an ABI PRISM cycler (Applied Biosystems) using a SYBR Green PCR kit from Stratagene and specific primers for CD83 and CCL4. Relative mRNA levels were determined by using included standard curves for each individual gene and further normalization to the housekeeping gene RPS9.

### Isolation of dendritic cells

For DC isolation, MLN were first cut into small pieces and then treated with 1 mg/ml collagenase type D (Roche) and 10 µg/ml DNase I type II (Sigma) diluted in PBS with 2% FCS and 2 mM EDTA. Enzymatic digestion was performed for 45 min at 37°C. The remaining tissue was mechanically minced and filtered through a 100 µm cell strainer. Cells were washed in PBS containing 2% FCS and 2 mM EDTA. Cell suspensions were incubated with α-CD11c MACS beads (Miltenyi Biotec). CD11c^+^ cells were positively selected on MACS columns according to manufacturer's instructions (Miltenyi Biotec).

### Proliferation assays

For CCL4 and CCL3 dependent proliferation assays 5×10^5^ splenocytes isolated from TCR-HA or CL4-TCR transgenic mice were stimulated with the cognate HA peptide in the presence of indicated concentrations of recombinant CCL4 or CCL3. Proliferation was measured by [^3^H]thymidine incorporation.

### Suppression assay

4×10^4^ HA-specific CD8^+^ T cells isolated from the MLN of CL4-TCR or VILLIN-HA/CL4-TCR transgenic mice were co-cultured with 4×10^4^ naive carboxyfluorescein succinimidyl ester (CFSE)-labeled HA-specific CD8^+^ (CL4-TCR) or CD4^+^ (TCR-HA) T responder cells in the presence of 4×10^5^ irradiated antigen-presenting cells (APCs) and the cognate HA peptide. At day 4 proliferation of responder cells was measured by loss of CFSE dye.

### *In vitro* induction of CD8^+^Foxp3^+^ T cells

2.5×10^5^ HA-specific CD8^+^ naïve T cells were cultured with 0.5×10^5^ MLN DCs, 0.1 µg/ml HA 512–520, 2 ng/ml human rTGF-β (R&D Biosystems), and 100 nM retinoic acid (RA) (Sigma-Aldrich). 50 U/ml human rIL-2 (eBioscience) was added to the cultures on day 2. On day 4, Foxp3 expression in CD8^+^ T cells was determined by staining with α-CD8 and α-Foxp3 antibodies.

## Results

### Intestinal antigen display promotes the induction of CD8^+^Foxp3^+^ T cells

**Figure 1:**
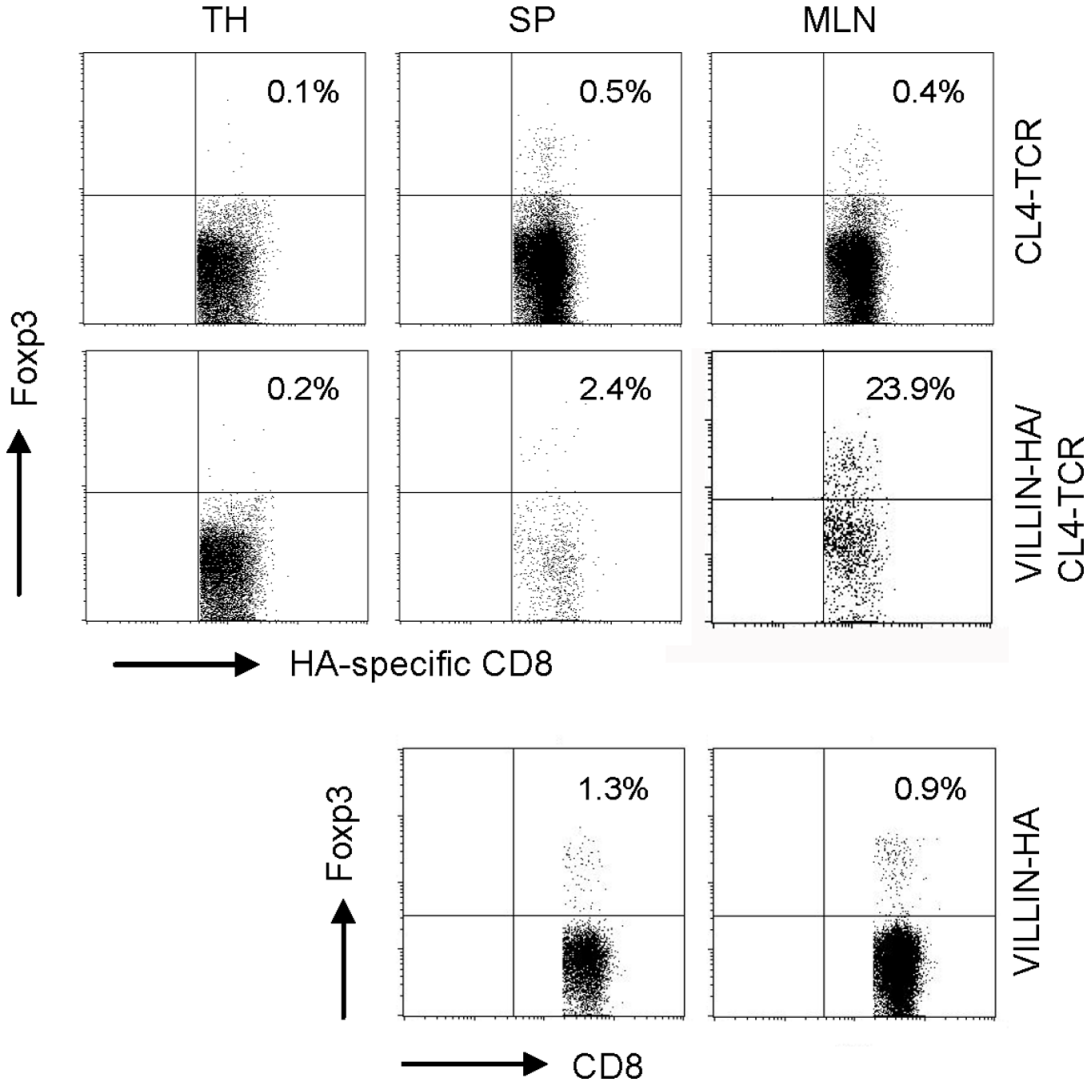
Induction of antigen-specific CD8^+^Foxp3^+^ T cells in vivo. Lymphocytes from the thymus (TH), spleen (SP) and mesenteric lymph nodes (MLN) of CL4-TCR, VILLIN-HA/CL4-TCR and VILLIN-HA transgenic mice were stained for the expression of CD8 and H-2K^d^IYSTVASSL and analyzed regarding the expression of Foxp3. Percentage of Foxp3^+^ T cells is indicated. One representative experiment out of four independent experiments with similar results is shown.

**Figure 2:**
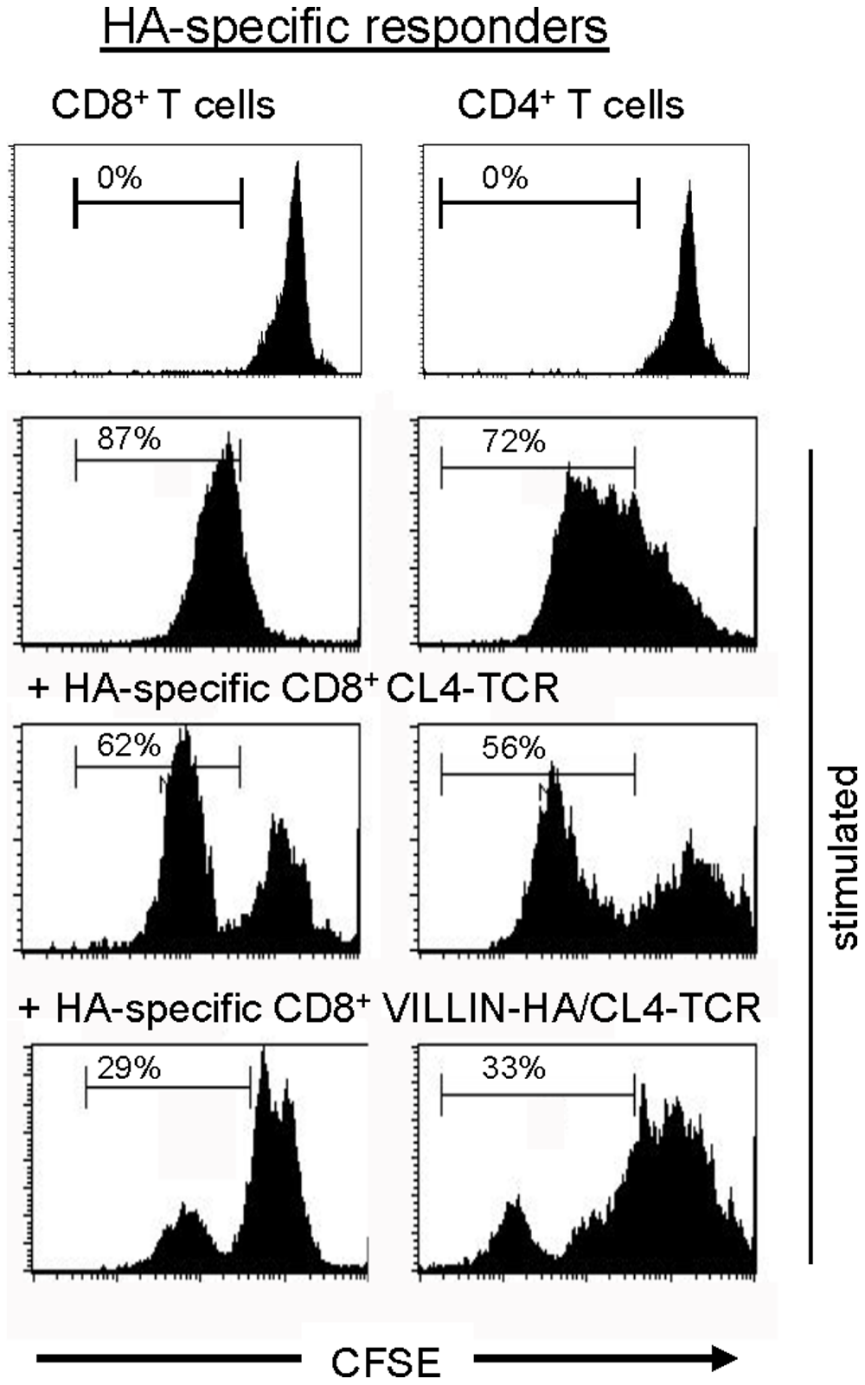
HA-specific CD8^+^ T cells from VILLIN-HA/CL4-TCR exhibit suppressive capacity. HA-specific CD8^+^ T cells were isolated from the MLN of CL4-TCR and VILLIN-HA/CL4-TCR transgenic mice by cell sorting and co-cultured with CFSE-labeled HA-specific CD4^+^ or CD8^+^ responder cells in the presence of the cognate peptide. Proliferation of responder cells was measured by loss of CFSE dye. Data shown are representative of three independent experiments.

We recently demonstrated that in VILLIN-HA/CL4-TCR transgenic mice chronic intestinal antigen exposure leads to the infiltration of HA-specific CD8^+^ T cells into the intestinal mucosa but without development of severe intestinal inflammation [10]. In the present study we further analyzed the infiltrating CD8^+^ T cells and demonstrated a partial induction of CD8^+^Foxp3^+^ T cells which was restricted to the periphery (Sp and MLN) and not detectable in the thymus (Figure 1). Characterization of HA-specific CD8^+^ T cells isolated from the MLN of VILLIN-HA/CL4-TCR transgenic mice reflected a reduced secretion of IFN-γ, TNF-α, and IL-6 after *in vitro* stimulation (Table 1). To analyze these antigen experienced HA-specific CD8^+^ T cells of VILLIN-HA/CL4-TCR transgenic mice in more detail the suppressive capacity *in vitro* was measured. For this purpose HA-specific CD8^+^ T cells were FACS-sorted by pentamer-staining (H-2K^d^IYSTVASSL^+^) from the MLN of control CL4-TCR and VILLIN-HA/CL4-TCR transgenic mice and co-cultured with naïve CFSE-labeled HA-specific CD8^+^ or CD4^+^ responder T cells in the presence of APCs and cognate HA peptide. Interestingly, HA-specific CD8^+^ T cells isolated from VILLIN-HA/CL4-TCR transgenic mice were able to suppress both CD8^+^ and CD4^+^ T cell proliferation *in vitro* (Figure 2).

**Table 1:** Detection of cytokine production by HA-specific CD8^+^ T cells from MLN of CL4-TCR and VILLIN-HA/CL4-TCR transgenic mice by Cytometric Bead Array.

Location	Mice	INF-γ (pg/ml)	TNF-α (pg/ml)	IL-6 (pg/ml)
**Mesenteric lymph node**	CL4-TCR	>5000	283±3	90±4
	VILLIN-HA/CL4-TCR	2000±320*	4±4*	35±3***

Transgenic CD8^+^ H-2K^d^IYSTVASSL^+^ T cells from the MLN of CL4-TCR and VILLIN-HA/CL4-TCR transgenic mice were sorted and stimulated *in vitro* with the HA512-520 peptide. Culture supernatants were analyzed for several cytokines using the cytokine bead array from BD. Cytokine quantities are depicted as pg/ml per 2.5×10^4^ CD8^+^ H-2K^d^IYSTVASSL^+^ T cells. Mean values of two independent experiments with two mice, respectively are depicted.

*p<0.05,

***p<0.001.

### Molecular characterization of peripheral induced CD8^+^ T_regs_

**Figure 3:**
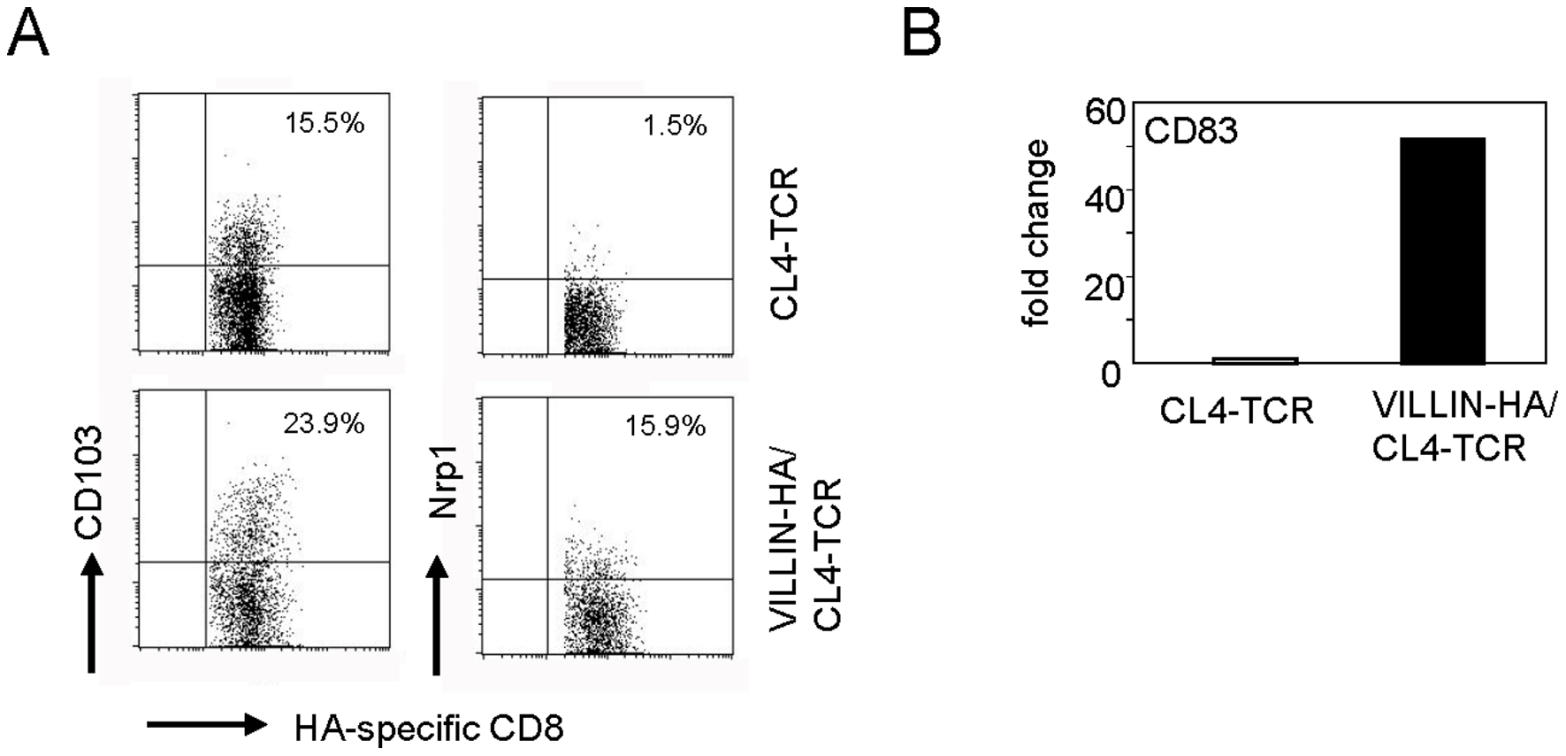
Phenotype of HA-specific CD8^+^ T cells from VILLIN-HA/CL4-TCR transgenic mice. (A) Cells isolated from the MLN of CL4-TCR and VILLIN-HA/CL4-TCR transgenic mice were stained for the expression of CD8, H-2K^d^ IYSTVASSL, CD103 and Nrp1. Dot plots represent the percentage of HA-specific CD8^+^ T cells expressing the indicated molecules. (B) HA-specific CD8^+^ T cells were isolated from the MLN of CL4-TCR and VILLIN-HA/CL4-TCR transgenic mice by cell sorting. CD83 expression was assayed by quantitative RT-PCR and normalized relative to expression of RPS9. Data shown are representative of three independent experiments.

To gain insights into the phenotype of induced antigen-specific CD8^+^ T_regs_, the phenotypical characteristics of these cells were analyzed at the molecular level by Affymetrix gene chips. For gene expression profiling, HA-specific CD8^+^ T cells were FACS-sorted by pentamer-staining from the MLN of CL4-TCR control mice and VILLIN-HA/CL4-TCR transgenic mice. To analyze whether CD4^+^ and CD8^+^ T_regs_ share common features, gene expression data were first evaluated for the expression of CD4^+^ T_reg_ specific marker molecules. Interestingly, HA-specific CD8^+^ T cells from VILLIN-HA/CL4-TCR transgenic mice with regulatory properties expressed a variety of genes that are specific for CD4^+^ T_regs_. A description of these genes is summarized in Table 2. Gene chip analysis in this study exhibited an upregulation of CD103 expression on HA-specific CD8^+^ T cells isolated from the MLN of VILLIN-HA/CL4-TCR transgenic mice. Furthermore, neuropilin 1 (Nrp1), programmed cell death 1 (Pdcd1), tumor necrosis factor receptor superfamily 9 (Tnfrsf9), lymphocyte-activation gene 3 (Lag3), cytotoxic T-Lymphocyte antigen 4 (CTLA-4) and CD83 were stronlgy upregulated by HA-specific CD8^+^ T_regs_. In contrast, IL7r, which is low expressed on CD4^+^ T_regs_, was also downregulated in HA-specific CD8^+^ T cells from VILLIN-HA/CL4-TCR transgenic mice in comparison to CD8^+^ T cells from CL4-TCR control mice. The validation of gene chip analysis was performed for selected genes by FACS staining (Figure 3A) and real-time RT PCR (Figure 3B).

**Table 2:** Differentially gene expression of T_reg_-associated genes by HA-specific CD8^+^ T cells isolated from MLN of CL4-TCR and VILLIN-HA/CL4-TCR transgenic mice.

Probe set	CL4-TCR		VILLIN-HA/CL4-TCR		VILLIN-HA/CL4TCR vs.CL4-TCR	Gene symbol
	Signal	Present or absent classified*	Signal	Present or absent classified*	Fold change	
1420692_at	12	A	114	P	**9.3**	Il2ra
1428735_at	950	P	2987	P	**3.1**	CD69
1425569_a_at	18	A	57	M	**3.1**	Slamf1
1460469_at	53	A	853	P	**16.1**	Tnfrsf9
1447541_s_at	594	P	4012	P	**6.7**	CD103
1449216_at	194	P	1424	P	**7.3**	CD103
1450357_a_at	34	A	105	P	**3.1**	Ccr6
1423466_at	1839	P	689	P	**−2.7**	Ccr7
1449925_at	784	P	334	P	**−2.3**	Cxcr3
1449911_at	5	A	269	P	**46.4**	Lag3
1448943_at	48	P	510	P	**10.4**	Nrp1
1449835_at	53	P	614	P	**11.5**	Pdcd1
1455439_a_at	379	P	3163	P	**8.3**	Lgals1
1419573_a_at	159	P	696	P	**4.4**	Lgals1
1420895_at	444	P	632	P	**1.5**	Tgfbr1
1443937_at	683	P	1707	P	**2.5**	Il2rb
1417597_at	1616	P	2414	P	**1.5**	CD28
1437025_at	624	P	1312	P	**2.1**	CD28
1433741_at	4	A	366	P	**89.5**	CD38
1434376_at	306	P	1726	P	**5.6**	CD44
1423760_at	320	P	1416	P	**4.4**	CD44
1419480_at	5440	P	1867	P	**−2.9**	CD62L
1448862_at	899	P	405	P	**−2.2**	Icam2
1452661_at	374	P	1477	P	**3.9**	Tfrc
1448575_at	5515	P	1358	P	**−2.8**	Il7r
1419334_at	16	A	449	P	**26.6**	Ctla4
1416111_at	26	A	810	P	**35.2**	CD83

### Differential gene expression of CCL4

**Figure 4:**
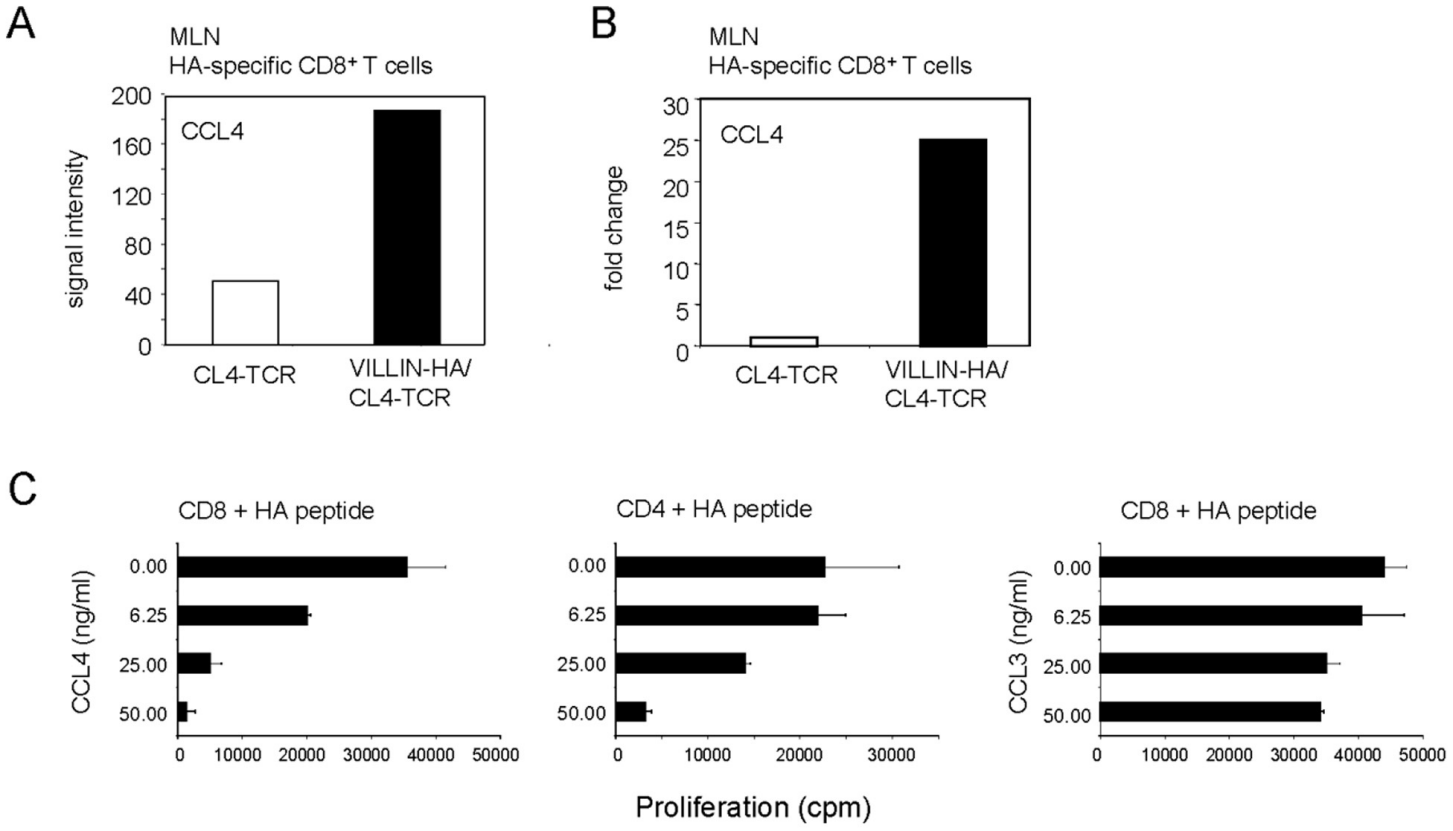
Specific expression of CCL4 by HA-specific CD8^+^ T cells from VILLIN-HA/CL4-TCR transgenic mice. (A) CD8^+^H-2K^d^IYSTVASSL^+^ were sorted from the MLN of CL4-TCR and VILLIN-HA/CL4-TCR transgenic mice. Affymetrix gene chip experiments were performed and analyzed for the expression of CCL4. Expression of CCL4 is indicated as signal intensity. (B) CD8^+^ H-2K^d^IYSTVASSL^+^ T cells were isolated from the MLN of CL4-TCR and VILLIN-HA/CL4-TCR transgenic mice by cell sorting. CCL4 expression was assayed by quantitative PCR and normalized relative to expression of RPS9. Data shown are representative of three independent experiments. (C) HA-specific CD4^+^ T cells (TCR-HA) and HA-specific CD8^+^ T cells (CL4-TCR) were stimulated with the corresponding antigen in the presence of indicated concentrations CCL4 or CCL3. Proliferation was measured by [^3^H]thymidine incorporation. One representative experiment out of three independent experiments is shown.

To get insights into the mechanism of antigen-specific CD8^+^ T_reg_-suppression, gene expression analysis of naïve HA-specific CD8^+^ T cells and HA-specific CD8^+^ T_regs_ were compared for the expression of genes which were exclusively expressed in HA-specific CD8^+^ T_regs_. Surprisingly, neither IL-10 nor TGF-β, molecules discussed in the context of CD4^+^ and CD8^+^ T_reg_ function were specifically expressed by *in vivo* induced CD8^+^ T_regs_ in our model. Screening for other secretory factors revealed CCL4 to be exclusively expressed by HA-specific CD8^+^ T cells from VILLIN-HA/CL4-TCR transgenic mice (Figure 4A). This was further confirmed by real-time RT PCR (Figure 4B). To investigate whether CCL4 is mechanistically involved in suppression, HA-specific CD4^+^ and CD8^+^ T cells were stimulated with the cognate HA peptides in the presence of varying concentrations of CCL4. As control varying concentrations of CCL3 were included into the experiment. Interestingly, CCL4 but not CCL3 was able to significantly inhibit proliferation of antigen-specific CD8^+^ and CD4^+^ T cells in a dose-dependent fashion (Figure 4C).

### Antigen-presentation by gut-associated DCs induces CD8^+^Foxp3^+^ T cells

**Figure 5:**
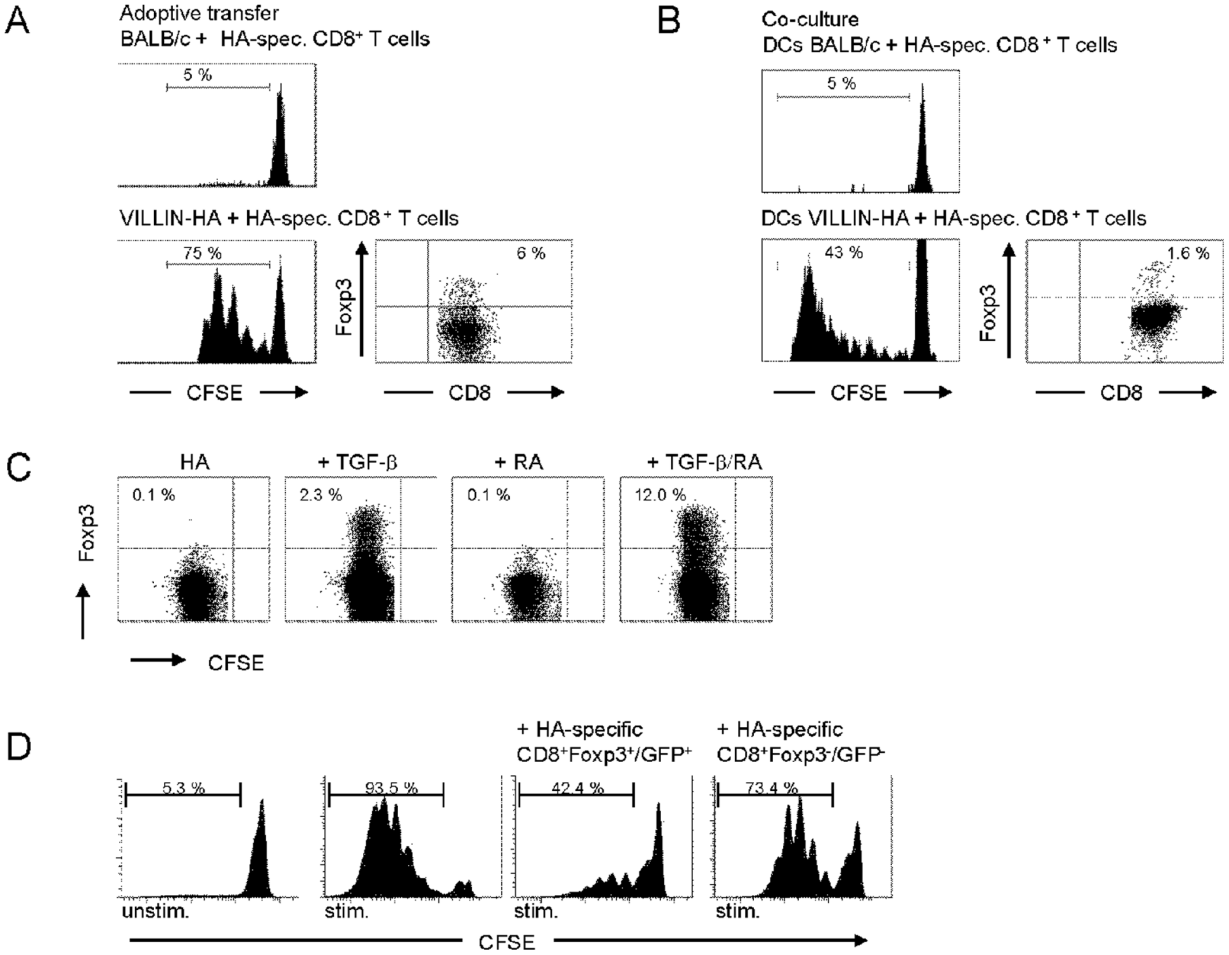
Peripheral induction of CD8^+^Foxp3^+^ T cells in vivo and in vitro. (A) CFSE-labeled HA-specific CD8^+^CD25^−^ T cells from CL4-TCR transgenic mice were adoptively transferred into VILLIN-HA and BALB/c recipient mice. At day 4 after adoptive transfer cells from the MLN of recipient mice were isolated and stained for the expression of CD8 and Foxp3. Histograms show proliferation of HA-specific CD8^+^ T cells by loss of CFSE dye, dot plot demonstrates the expression of Foxp3 in proliferating CD8^+^ T cells. (B) DCs from MLN of VILLIN-HA and BALB/c mice were co-cultured with CFSE-labeled HA-specific CD8^+^CD25^−^ T cells from CL4-TCR transgenic mice for 5 days. Cells were stained for the expression of CD8 and Foxp3. Histograms show proliferation of HA-specific CD8^+^ T cells by loss of CFSE-dye, dot plot demonstrates the expression of Foxp3 in proliferating CD8^+^ T cells. (C) CFSE-labeled HA-specific CD8^+^CD25^−^ T cells from CL4-TCR transgenic mice were co-cultured with MLN DCs from BALB/c mice and exogenous HA peptide for 4 days. Where indicated, cells were supplemented with 2 ng/ml human rTGF-β or 100 nM RA. Cells were stained for the expression of CD8 and Foxp3. Gating on proliferating CD8^+^ T cells, the expression of Foxp3 vs CFSE fluorescence intensity is demonstrated. Data shown are representative of three independent experiments. (D) For in vitro T cell suppression assays, HA-specific CD8^+^ T cells were separated into CD8^+^Foxp3^−^/GFP^-^ and CD8^+^Foxp3^+^/GFP^+^ T cells by FACS on CD8 and GFP expression. Sorted T cells were co-cultured with freshly isolated CFSE-labeled HA-specific CD4^+^CD25^−^ responder T cells and APCs, and stimulated with the cognate HA peptides. Histograms show proliferation of responder T cells as determined by loss of CFSE dye. Data from two independent experiments are shown.

For the CD4^+^ T cell subset it was demonstrated that naturally occurring CD4^+^CD25^+^Foxp3^+^ T_regs_ are the most prominent CD4^+^ T cells with immune modulatory capacity. In contrast, in a healthy host such naturally occurring CD8^+^Foxp3^+^ T cells are very rare. To analyze whether the induction of Foxp3 in HA-specific CD8^+^ T cells is predetermined in the thymus rather than in the periphery, adoptive transfer experiments into VILLIN-HA recipient mice were performed. Thus, CFSE-labeled HA-specific CD8^+^Foxp3^−^ T cells were intravenously injected into VILLIN-HA recipient mice. At day 4 after transfer, cells from the MLN were isolated and analyzed for the proliferation of HA-specific CD8^+^ T cells. In contrast to the adoptive transfer into BALB/c mice, HA-specific CD8^+^ T cells in VILLIN-HA transgenic mice exhibited a strong proliferative response as demonstrated by loss of CFSE dye. Gating on proliferating cells revealed that antigen stimulation in gut-associated tissues *in vivo* leads to the peripheral induction of CD8^+^Foxp3^+^ T cells as depicted in Figure 5A.

Recently, we could demonstrate that gut-associated DCs isolated from VILLIN-HA transgenic mice are able to present the endogenous HA antigen and to induce the differentiation of HA-specific CD4^+^Foxp3^+^ T cells *in vitro* [18]. To investigate the role of gut-associated DCs for the induction of CD8^+^Foxp3^+^ T cells, DCs isolated from the MLN of VILLIN-HA transgenic mice were co-cultured with CFSE-labeled HA-specific CD8^+^Foxp3^−^ T cells *in vitro*. Whereas DCs isolated from VILLIN-HA transgenic mice predominantly induced the proliferation of CD8^+^Foxp3^−^ T cells, a small fraction of CD8^+^Foxp3^+^ T cells was generated *in vitro* (Figure 5B). To determine whether RA in combination with TGF-β could be responsible for the conversion of naïve CD8^+^Foxp3^−^ T cells into CD8^+^Foxp3^+^ T cells DCs from the MLN of BALB/c mice were isolated and co-cultured with HA-specific CD8^+^Foxp3^−^ T cells from CL4-TCR transgenic mice in the presence of HA peptide, or a combination with RA or TGF-β. Interestingly, co-culture of HA-specific CD8^+^ T cells with DCs in the presence of HA peptide or HA peptide in combination with RA does not lead to a clear conversion into CD8^+^Foxp3^+^ T cells (Figure 5C). In contrast, co-culture in the presence of HA peptide and TGF-β induces the conversion of a small population of CD8^+^Foxp3^+^ T cells (2.3%), which is further increased by adding HA peptide, TGF-β and RA (12%).

To gain further insight into the immunosuppressive function of TGF-β/RA-induced CD8^+^Foxp3^+^ T cells and to allow accurate separation of Foxp3^+^ and Foxp3^−^ CD8^+^ T cells, we used CL4-TCR/Foxp3/GFP transgenic reporter mice, in which GFP expression identifies the Foxp3^+^ T cell population. HA-specific CD8^+^Foxp3^−^ T cells were stimulated in the presence of TGF-β and RA. HA-specific CD8^+^Foxp3^+^/GFP^+^ and CD8^+^Foxp3^−^/GFP^−^ T cells were sorted by FACS and co-cultured with naïve CFSE-labeled HA-specific CD4^+^ responder T cells in the presence of APCs and the cognate HA peptides. At day 6 after stimulation, proliferation of responder cells was measured by the loss of CFSE dye. As shown in Figure 5D TGF-β/RA-treated HA-specific CD8^+^Foxp3^+^ T cells markedly suppressed the proliferation of CD4^+^ responder T cells which demonstrated the regulatory activity of CD8^+^Foxp3^+^ T cells *in vitro*.

## Discussion

Like most autoimmune syndromes, inflammatory bowel disease is considered to represent an uncontrolled immune response in genetically predisposed hosts. Gut luminal antigens have attracted great attention, and the current paradigm proposes that interactions of such bacteria with the host's epithelial cells and the mucosal immune system eventually results in continuous microbial antigenic stimulation and associated tissue damage [19]. T_regs_ are believed to be crucial in adjusting response thresholds to microbial antigen as well as modulating tissue damaging immune reactions [20]. Limited information is available about the phenotypic and functional characteristics of such regulatory/suppressor T cells in the intestinal mucosa. The most prominent population of T_regs_ belongs to the CD4^+^ T cell subset. In this subset T_regs_ are classified in naturally occurring CD4^+^CD25^+^Foxp3^+^ T_regs_ and those that can be induced in the periphery. There is, however, evidence that CD8^+^ T cells may also harbour suppressive potential. This warrants further exploration. Allez et al. have generated CD8^+^ T_regs_ by stimulating peripheral blood T cells with irradiated allogeneic intestinal epithelial cells [7]. Phenotypic markers of such CD8 T_regs_ included CD101 and CD103 but no expression of Foxp3. In addition, Ménager-Marcq et al. have demonstrated that CD8^+^CD28^−^ but not CD8^+^CD28^+^ T cells freshly isolated from the spleen or the gut efficiently prevented the development of colitis [8]. In our VILLIN-HA/CL4-TCR transgenic mouse model we could demonstrate that chronic antigen stimulation in the gut and the gut-associated lymph nodes leads to the peripheral induction of antigen-specific CD8^+^Foxp3^+^ T cells with inhibitory capacity for both CD4 and CD8 T cell proliferation. In contrast to the former described Foxp3 independent CD8^+^ T_regs_ the phenotype of these CD8^+^Foxp3^+^ T cells is strikingly similar to naturally occurring CD4^+^Foxp3^+^ T_regs_ (Table 2). Most notably, they specifically express cell-surface molecules like CD103, Nrp1, Pdcd1 and Tnfrsf9. CD103 is the receptor for E-cadherin and has initially been described as present on the cell surface of CD8^+^ lymphocytes localized in the intestine. An important function of this molecule is to direct lymphocytes to E-cadherin-expressing epithelial cells [21]. It was demonstrated that CD103 defines a subset of human alloantigen-induced CD8^+^ T cells that posses the functional features of regulatory T cells [5]. Interestingly, allostimulated CD8^+^CD103^+^ T cells lacked Foxp3, CD25, LAG3, CTLA4, and GITR expression suggesting that these cells belong to another subtype of CD8^+^ T_regs_. Very recently, a subpopulation of CD8^+^CD25^+^Foxp3^+^ suppressive T cells in patients suffering from colorectal cancer was identified [9]. In these patients CD8^+^CD25^+^Foxp3^+^ T cells were detectable in the blood and more prominently in the colorectal cancer tissue with a phenotype closely resembling the CD8^+^Foxp3^+^ T cells from our transgenic mouse model. It is likely that the intestine enriched with large amounts of TGF-β serves as a specific site for CD8^+^Foxp3^+^ T cell induction.

TGF-β is a regulatory cytokine that has a strong impact on the induction and function of T_regs_. In response to local factors, gut lamina propria cells and intestinal epithelial cells release abundant TGF-β[22]. It was demonstrated that activation of naive CD4^+^CD25^−^ T cells in the presence of TGF-β *in vitro* can induce Foxp3 expression [23], [24]. Most interesting in this context was the observation that DCs from gut origin can markedly enhance TGF-β induced conversion of CD4^+^ T cells into the Foxp3^+^ phenotype *in vitro* dependent on RA [12], [13]. RA, the key metabolite of Vitamin A, seems to play a predominant role in the homeostasis and homing of lymphoid populations of the gut-associated lymphoid tissue (GALT). It is synthesized in abundance by intestinal gut and gut-associated DCs [12], [25], induces the specific gut-homing molecules CCR9 and α_4_β_7_ integrin on T cells, and also promotes GALT-related functions in B cells [25]. RA's important role in controlling Foxp3 expression mediated by TGF-β also suggests that the GALT has evolved a specific system to maintain a balanced symbiosis between the gut flora and the immune system [12], [13], [26], [27]. Intriguingly, we could demonstrate that the potential of gut-associated DCs to convert naive T cells into Foxp3^+^ T cells is also true for CD8^+^ T cells. Detailed characterization of induction of CD8^+^Foxp3^+^ T_regs_ revealed the dependency on TGF-β and RA for this process. *In vitro* and *in vivo* experimental systems investigating polyclonal populations of CD8^+^ T_regs_ have led to the description of several apparently distinct mechanisms of immune regulation. It was demonstrated that CD8^+^ T_regs_ may act by killing of the target cells, negative signaling directly on the target cells or on APCs and the secretion of soluble factors, such as immunosuppressive cytokines like IL-10, TGF-β or CCL4 [3], [28], [29]. One explanation for these multiple mechanisms could be the existence of separate subsets of CD8^+^ T_regs_ that use different mechanisms. For antigen-specific CD8^+^ T_regs_ detected in the VILLIN-HA/CL4-TCR transgenic mice we demonstrated the specific expression of CCL4. Although CCL4 is commonly regarded as proinflammatory, several lines of evidence are compatible with an additional, regulatory role for CCL4. We could demonstrate that CCL4 in contrast to CCL3 inhibited the proliferation of stimulated antigen-specific CD4^+^ and CD8^+^ T cells *in vitro*. These results are well in line with a study that demonstrated that the stimulation with Bacillus Calmette-Guérins results in the differentiation of CD8^+^CD25^+^Lag3^+^Foxp3^+^ T cells, which suppress T cell proliferation partly through the secretion of CCL4 [3].

In conclusion, we describe the existence of a regulatory CD8^+^ T cell subset in the gut-associated tissue. These CD8^+^ T cells have suppressive function and accumulate in the mesenteric lymph node. They can be induced by gut-associated DCs dependent on TGF-β and RA. However, CD8^+^Foxp3^+^ T_regs_ represent a small fraction of CD8^+^ T cells *in vivo*, therefore it is important to determine their relevance in more detail.
